# Editorial: Drug development of herbal medicines: Regulatory perspectives

**DOI:** 10.3389/fphar.2022.989934

**Published:** 2022-11-10

**Authors:** Anna Rita Bilia, Pulok Kumar Mukherjee, Adolfo Andrade-Cetto, Chandra Kant Katiyar, Sitesh C. Bachar, Motlalepula Gilbert Matsabisa, Subhash C. Mandal

**Affiliations:** ^1^ Department of Chemistry “Ugo Schiff”, University of Florence, Firenze, Italy; ^2^ Institute of Bio-Resources and Sustainable Development (IBSD), Imphal, India; ^3^ National Autonomous University of Mexico, México City, Mexico; ^4^ CEO Health Care (Technical), Emami Ltd., Kolkata, India; ^5^ Department of Pharmacy, Faculty of Pharmacy, University of Dhaka, Dhaka, Bangladesh; ^6^ University of the Free State, Bloemfontein, South Africa; ^7^ Society for Ethnopharmacology India, Kolkata, India

**Keywords:** herbal medicine, regulatory perspectives, TCM, botanicals, ayurveda

The global trade of medicinal plants and their derivatives was estimated at US$33 billion in 2014, and the World Health Organization has estimated that it will increase to US$50 trillion by 2050. Different regulatory frameworks and categories at the national and regional levels describe medicinal plants either as mainstream therapy or as complementary and alternative medicines. The resulting complex terminology has seen medicinal plants classified as medicines (Australia), herbal medical products (European Union), botanicals (United States), and natural health products (Canada). In China, there is a distinction between traditional Chinese medicine (TCMs) and natural medicinal products. In India, traditional medicine is separated into three systems: Ayurveda, Unani, and Siddha. In Japan, Kampo medicines are classified as pharmaceutical drugs, and in Thailand, as part of the primary health care system. Many national health authorities have established guidelines and regulations concerning the quality, efficacy, and safety profiles of these products. Five papers are included in this Research Topic, all of which concern these three fundamental aspects of the health properties of herbal medicines.

The paper by Chen et al. discusses the need to develop quality control systems to evaluate TCMs by assessing the quality control measures used for Glehniae Radix, a medicinal plant, along its value chains (VCs). Glenhae Radix was chosen as a “model” plant material due to its constantly increasing global demand, especially in Asian countries. Previous studies have shown that the production and processing methods of different VCs impact the quality of the medicinal materials. Four years of field and market research were conducted for the study, including interviews with stakeholders in the VCs. Different types of VCs were integrated and further analyzed. The authors found that vertical integration in the VCs could guarantee not only benefits for the growers but also the traceability of the medicinal materials, which further guarantees their quality.

The study by Rujanapun et al. explores the effects of special Thai oolong tea steamed with selected Thai botanical drugs, with a focus on the effects of the botanical drugs on biological processes, namely hypoglycemic activity. Interestingly, among all the varieties studied, the tea made of oolong tea leaves steamed with Indian gooseberry (*Phyllanthusemblica*) exhibited the best activity in *vitro* assays, i.e., antioxidant, anti-inflammation, and anti-adipogenesis activity, enzyme inhibition, and, in particular, the inhibition of glucose uptake and consumption by adipocytes and skeletal muscle. In addition to the tea catechins, these effects could be caused by the presence of flavonoid compounds contributed by the Thai botanical drugs.

The paper by Wan-Tong et al. aimed to explore the safety and efficacy of Yuanjiang decoction, a traditional Chinese medicinal prescription, for symptomatic bradyarrhythmia on a compassionate-use basis. Eligible participants were recruited and treated with 200 ml of Yuanjiang decoction (composed of six Chinese herbal medicines) twice daily for 16 weeks. Analyses were done with the intention-to-treat approach. The primary outcome measure was the proportion of participants who achieved a favorable treatment outcome at 16 weeks. A total of 184 patients were included. After 16 weeks of treatment, 12 participants were lost to contact and 21 participants were terminated from the study, for a drop-out rate of 17.93%. The most common treatment-related adverse events were xerostomia (6.52%), constipation (6.45%), and sleepiness (3.26%). The proportion of participants with a favorable treatment outcome was 65.22% at 4 weeks, 59.78% at 8 weeks (OR: 1.11, 95% CI: 0.71–1.73), 61.41% at 12 weeks (OR: 1.16, 95% CI: 0.92–1.45), and 60.87% at 16 weeks (OR: 1.15, 95% CI: 0.98–1.35). In the multifactor regression analysis, a favorable treatment outcome at 16 weeks was significantly associated with completing at least 8 weeks of treatment (OR: 2.053, 95% CI: 1.064–3.560), while an unfavorable treatment outcome was significantly associated with having an atrioventricular block (OR: 0.255, 95% CI: 0.083–0.784), being a current smoker (OR: 0.343, 95% CI: 0.027–0.487), or experiencing syncope in the month before treatment (OR: 0.321, 95% CI: 0.114–0.904). In conclusion, the results showed encouraging treatment outcomes for Yuanjiang decoction, without serious adverse events.

The paper by Gao et al. investigated Huoxiang Zhengqi (HXZQ), an extensively used TCM formula that targets gastrointestinal disorders such as gastroenteritis by regulating the intestinal microbiome/immune microenvironment. In this study, the effects of HXZQ on the gut microbiome of healthy adults were investigated for the first time, and a mice model of antibiotic-induced gut dysbiosis was applied for verification. The results revealed that HXZQ exhibited mild and positive effects on bacterial diversity and gut microbiome composition in a healthy state. In states of dysbiosis, HXZQ significantly restored bacterial diversity and increased the abundance of *Bacteroidetes*. In the antibiotic-induced mice model, HXZQ markedly revived deficient gut microbial compositions. At the genus level, the bacteria that responded most strongly and positively to HXZQ were *Bifidobacterium* in healthy adults and Muribaculaceae, *Lactobacillus*, and *Akkermansia* in mice. In contrast, the abundance of *Blautia* in healthy adults, and of *Enterococcus* and *Klebsiella* in mice, showed an inverse association with HXZQ administration. The authors speculated that HXZQ might exhibit an anti-inflammatory effect in mice by regulating the concentration of interleukin-6 in plasma while causing no significant changes in the colon tissue structure. Ultimately, their results elucidated the modulatory effects of HXZQ on the structure of the gut microbial community and confirmed its safety for daily use.

The article by Xu et al. aimed to investigate whether Gentiopicroside (GPS), a natural compound with anti-inflammatory properties, inhibits renal tubulointerstitial fibrosis (TIF) in diabetic db/db mice and in high glucose (HG)–stimulated renal tubular epithelial cells. The *in vivo* results showed that GPS effectively improved glycolipid metabolism disorder, renal dysfunction, and TIF. Specifically, GPS treatment reversed the abnormal expressions of epithelial–mesenchymal transition marker proteins, including elevated *α*-smooth muscle actin and vimentin and decreased E-cadherin, in the kidneys of db/db mice. Meanwhile, GPS treatment also inhibited expression of the proteins angiotensin II–type 1 receptor (AT1R) and CK2α, as well as the activation of the NF-κB pathway. Moreover, the above effects of GPS were also observed *in vitro* in HG-stimulated NRK-52E cells; these effects were independent of its effects on glucose and lipid lowering, but were reversed by AT1R over-expression.

Although the regulatory aspects of herbal medicines must be of major concern worldwide, we received only a small amount of manuscripts; we therefore think that this topic needs further attention in the near future. The development and evaluation of herbs and herbals are controlled and implemented through various agencies in different countries. The major challenges facing the development and promotion of these products include chemo-profiling, safety evaluations, quality control, and effective regulatory guidelines. However, these circumstances offer unique opportunities for researchers and those in the pharmaceutical industry to enhance drug discovery and development. Various strategic areas in medicinal plant research are currently being considered In order to revitalize herbal medicine and bring it in line with modern medicine. Over the past decades, public interest in natural therapies—namely, herbal medicine—has increased dramatically in both developing and industrialized countries. The traditional use of medicinal plants needs to be systematically investigated and standardized to ensure quality, safety, and efficacy in the development of herbal medicine. These efforts should be popularized to include a greater amount of stakeholders, as well as the general public, and to raise awareness regarding the quality and safety of ethnomedical products with interdisciplinary and transdisciplinary approaches.

